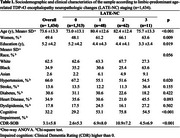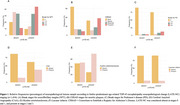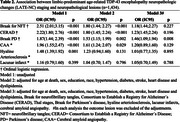# Association of LATE‐NC with advanced neuropathological lesions: a population‐based autopsy study

**DOI:** 10.1002/alz70855_100510

**Published:** 2025-12-23

**Authors:** Caroline Matos Silva, Alberto Fernando Oliveira Justo, Renata Elaine Paraizo Leite, Vitor Ribeiro Paes, Lea T. Grinberg, Carlos Augusto Pasquallucci, Eduardo Ferrioli, Ricardo Nitrini, Claudia Kimie Suemoto

**Affiliations:** ^1^ University of São Paulo Medical School, São Paulo, São Paulo, Brazil; ^2^ University of Sao Paulo Medical School, São Paulo, Brazil; ^3^ Memory & Aging Center, Department of Neurology, University of California in San Francisco, San Francisco, CA, USA

## Abstract

**Background:**

Limbic‐predominant age‐related TDP‐43 encephalopathy neuropathological change (LATE‐NC) is characterized by amnestic dementia, primarily affects adults over 80 and often coexists with other neuropathological lesions. We aimed to investigate the frequency of other neuropathological lesions and their associations with LATE‐NC.

**Method:**

This cross‐sectional study analyzed a population‐based sample of 1,434 Brazilians from the Biobank for Aging Studies at the University of São Paulo Medical School. Neuropathological data were collected for Braak for Parkinson's Disease (Braak for PD), Braak for neurofibrillary tangles (Braak for NFT), amyloid‐β burden (CERAD), LATE‐NC, lacunar infarcts, cerebral amyloid angiopathy, and hyaline arteriolosclerosis. Ordinal logistic regression evaluated the relationship between LATE‐NC and the staging of other neuropathological lesions. LATE‐NC was classified into four stages (0, 1, 2, 3). For descriptive analyses, LATE‐NC was dichotomized into binary categories: absent (stages 0–1) and present (stages 2–3). Models were adjusted for sociodemographic factors, clinical variables (hypertension, diabetes, heart disease, stroke, dyslipidemia and cognitive impairment), and neuropathological lesions (Braak for NFT, CERAD, Braak for PD, lacunar infarcts, hyaline arteriolosclerosis, and cerebral amyloid angiopathy). In each analysis, the outcome lesion was excluded from the adjustments.

**Result:**

Sociodemographic and clinical variables varied significantly across participants at different LATE‐NC stages (Table 1). Participants in LATE‐NC stages 1 and 2 exhibited a higher frequency of advanced neuropathological changes compared to those without LATE‐NC, while participants in stage 3 showed lower frequencies, likely due to the smaller sample size. LATE‐NC as a binary variable demonstrated an overall higher frequency of all neuropathological lesions associated with LATE‐NC (Figure 1). Advanced stages of Braak for NFT, Braak for PD, CERAD scores, cerebral amyloid angiopathy (CAA), and hyaline arteriolosclerosis were more prevalent among cases with LATE‐NC. Adjusted analyses for sociodemographic and clinical factors along with other neuropathological lesions, demonstrated a significant association only between LATE‐NC presence and advanced Braak PD stages (OR = 1.46, 95% CI = 1.09–1.94, *p* = 0.008).

**Conclusion:**

Higher stages of LATE‐NC were associated with an increased frequency of neuropathological lesions. Only Lewy body pathology was independently associated with LATE‐NC in adjusted analyses for sociodemographic, clinical, and neuropathological variables.